# Development of three-dimensional canine hepatic tumor model based on computed tomographic angiography for simulation of transarterial embolization

**DOI:** 10.3389/fvets.2023.1280028

**Published:** 2024-01-30

**Authors:** Miju Oh, Jiyoung Ban, Yooyoung Lee, Minju Lee, Sojin Kim, Uhjin Kim, Jiwoon Park, Jaepung Han, Jinhwa Chang, Byungjin Kim, Hyeongrok Yun, Namsoon Lee, Dongwoo Chang

**Affiliations:** ^1^Section of Veterinary Imaging, Veterinary Medical Center, College of Veterinary Medicine, Chungbuk National University, Cheongju, Republic of Korea; ^2^Korea Animal Medical Center, Cheongju, Republic of Korea; ^3^Bon Animal Medical Center, Suwon, Republic of Korea; ^4^SKY Animal Medical Center, Cheonan, Republic of Korea

**Keywords:** hepatocellular carcinoma, transarterial embolization, 3D-printed canine hepatic artery model, transarterial chemoembolization, intervention

## Abstract

**Introduction:**

Transarterial embolization (TAE) is one of the treatment options for liver masses that are not suitable for surgery and they have been applied in veterinary medicine for about 20 years, but surgical resection is considered as the first treatment option, and only a few case reports and articles about TAE in dogs have been published. Although understanding of vascular anatomy for the procedure is important, previous studies lack of the information about hepatic artery anatomy in small and toy-breed dogs. Due to the introduction of 3D print in veterinary medicine, it is now possible to make 3D models for preoperative planning. The purpose of this study is to understand the hepatic arterial vascular structure of various sizes and breeds of dogs, and to develop 3D-printed canine artery models with and without hepatic tumors to simulate TAE procedure.

**Methods:**

CT images of a total of 84 dogs with normal hepatic arteries were analyzed, and the mean value and standard deviation of body weight, celiac artery size, and hepatic artery size were 6.47 ± 4.44 kg, 3.28 ± 0.77 mm, and 2.14 ± 0.43 mm, respectively.

**Results:**

It was established that type 2-2-1, which has two separate hepatic branches—the right medial and left branch and the right lateral branch that runs to the right lateral lobe and caudate process—is the most prevalent of the hepatic artery branch types, as it was in the previous study. The review of 65 CT images of dogs with hepatic tumors showed that 44.6% (29/65) had multifocal lesions in multiple lobes, for which TAE can be recommended.

**Discussion:**

Based on the result, a 3D model of the normal canine hepatic artery and the hepatic tumor was made using one representative case from each group, and despite the models having some limitations in reflecting the exact tactile and velocity of blood vessels, TAE procedure was successfully simulated using both models.

## 1 Introduction

Unlike normal blood flow into the hepatic parenchyma, which is mostly derived from the portal veins (1), about 90% of blood flow into hepatic tumors depends on the hepatic artery (2). Using the vasculature and morphologic characteristic of the tumor, in human patients who do not meet the surgical indication or wait for liver transplantation, transarterial embolization (TAE) is performed to prevent blood flow into the tumor, or transarterial chemoembolization (TACE) is performed to increase local chemotherapy concentrations and dwell times within the tumor and reduce subsequent systemic toxicities (3, 4).

Transarterial embolization (TAE) has also been introduced in veterinary medicine about 2 decades ago (5). However, the most common primary liver tumors in dogs are hepatocellular masses and the most common gross morphology of them is massive type which means a solitary lesion in a single liver lobe (6, 7), which the prognosis is known to be good when complete resection is performed (6, 7), surgery is still chosen as the first-line treatment (8). But for the multifocal lesions affecting two or more hepatic lobes, the surgical resection becomes difficult even though the size of the mass is small (7, 9–12). Also, the efficacy of routine systemic chemotherapy is limited, therefore when multifocal lesions are observed in multiple lobes, the prognosis is known to be poor (13). Even though only a single hepatic mass exists, surgical treatment is not amenable when the tumor adhesion to other structures such as the diaphragm, large blood vessels, and other adjacent organs, traditional systemic chemotherapy can be attempted but only systemic chemotherapy without surgical resection is usually unsatisfied due to the resistance of tumor cells to chemotherapeutic drugs (11). In these cases, TAE can be an effective treatment for dogs with unresectable liver tumors (5, 10, 14–16), but since the liver consists of several lobes, it requires lots of practice for accurate procedures in real patients.

For TAE procedures, it is also important to understand the vascular structure of the hepatic artery. According to previous studies about the hepatic vascular system in dogs (17–20), types of the hepatic artery can be classified according to the number of branches ramified from the main hepatic artery, and in the most recent study, the most common type was division into two main branches (18). In another study, the average diameter of the celiac artery was 5.1 mm and the hepatic artery was 4.05 mm in normal beagle dogs (20). However, the weight of the dogs included in these studies was at least 8 kg and up to 30 kg (17–20), and the information about the characteristics of the hepatic artery in toy and small breed dogs is insufficient.

In recent veterinary medicine, 3D printing has been widely used for surgical planning, education, and even for the manufacture of graft substitutes (21–24). Previous articles introduced 3D models of animal livers, such as 3D vascular and biliary models of feline liver (25) and 3D liver vessel models for intrahepatic portosystemic shunt (IHPSS) surgery in dogs (26). In human medicine, 3D print models are also used in interventional therapy, there are some case reports and articles about hollow 3D printed models for endovascular interventions like coiling of splenic artery aneurysm, transcatheter valve replacement, and for bronchoscopy (27–29).

In this study, we expected the manufacture of the 3D print model of dogs for practicing interventional treatment as in human medicine, the TAE procedures on hepatic masses. We aimed to make two models, the normal liver model to become experienced with normal anatomy before going into the complicated vascular anatomy of masses and the hepatic mass model for actual practice of hepatic mass. We analyzed the hepatic artery structure of dogs with normal livers of various breeds and sizes to reflect the hepatic artery anatomy from small and toy breeds to large breed dogs to normal hepatic artery model and collected the CT angiography data of dogs with hepatic mass for hepatic mass model.

## 2 Materials and methods

### 2.1 Animals

The cases for the study were recruited and analyzed retrospectively from four veterinary medical centers: Chungbuk National University Veterinary Teaching Hospital, Korea Animal Medical Center, Bon Animal Medical Center, and Cheonan SKY Animal Medical Center. Following the analysis results of the cases, the optimal cases were selected to manufacture the 3D models. Between January 2016 and July 2022, dogs underwent triple-phase abdomen computed tomography (CT) including overall liver area, and had medical records that consisted of at least breed, weight, and reasons for CT were included. The dogs were divided into two groups, dogs with no lesions in liver at both pre- and post-contrast images and without problems of liver were classified as normal hepatic artery group, and dogs with liver lesions were classified as hepatic mass group. We included the cases with the arterial phase for the volume rendering, and the arterial phase was acquired by bolus tracking and the cases with inappropriate arterial phase were excluded, such as contamination of hepatic and portal veins by late exposure or the insufficient enhancement of hepatic artery by early exposure, or artifacts such as beam hardening and motion artifact affected the liver area.

### 2.2 CT image analysis

The CT examinations were performed using three different scanners: two 16-slice helical CT scanner (Revolution ACT, GE Medical Co., Milwaukee, WI, United States), a 16-slice helical CT scanner (SOMATOM Scope; SIEMENS, Tokyo, Japan), and a 64-slice helical CT scanner (Aquilion 64CFX, Toshiba Medical Systems, Otawara, Japan). CT scans were performed under general anesthesia and the slice thickness of three CT scanners was 1.0–2.0 mm with a pitch of 0.75–1.5. The scanning parameters were 100–150 kV tube voltage, 100–200 mA, 250–500 mm field of view, and 512 × 512 matrix. All images were evaluated using DICOM viewer (RadiAnt DICOM Viewer 2022.1.1; Medixant, Poznan, Poland). We made three-dimensional volume rendering using the images of arterial phase to analyze the branching pattern of the hepatic artery, and the measurement of hepatic artery diameters and the characteristics of liver lesions were analyzed in the transverse image. The evaluations were performed by two graduate students (MO and YL) of veterinary medicine at CBNU-VTH, and the disagreements were resolved through consensus.

### 2.3 Normal hepatic artery group

Dogs underwent CT scans for problems unrelated to the liver and no significant lesions observed in hepatic parenchyma were included in the normal hepatic artery group, and dogs with lesions causing displacement or compression of hepatic parenchyma and hepatic vessels such as large masses originated from adjacent organs and excessive ascites were also excluded.

**Figure 1:**
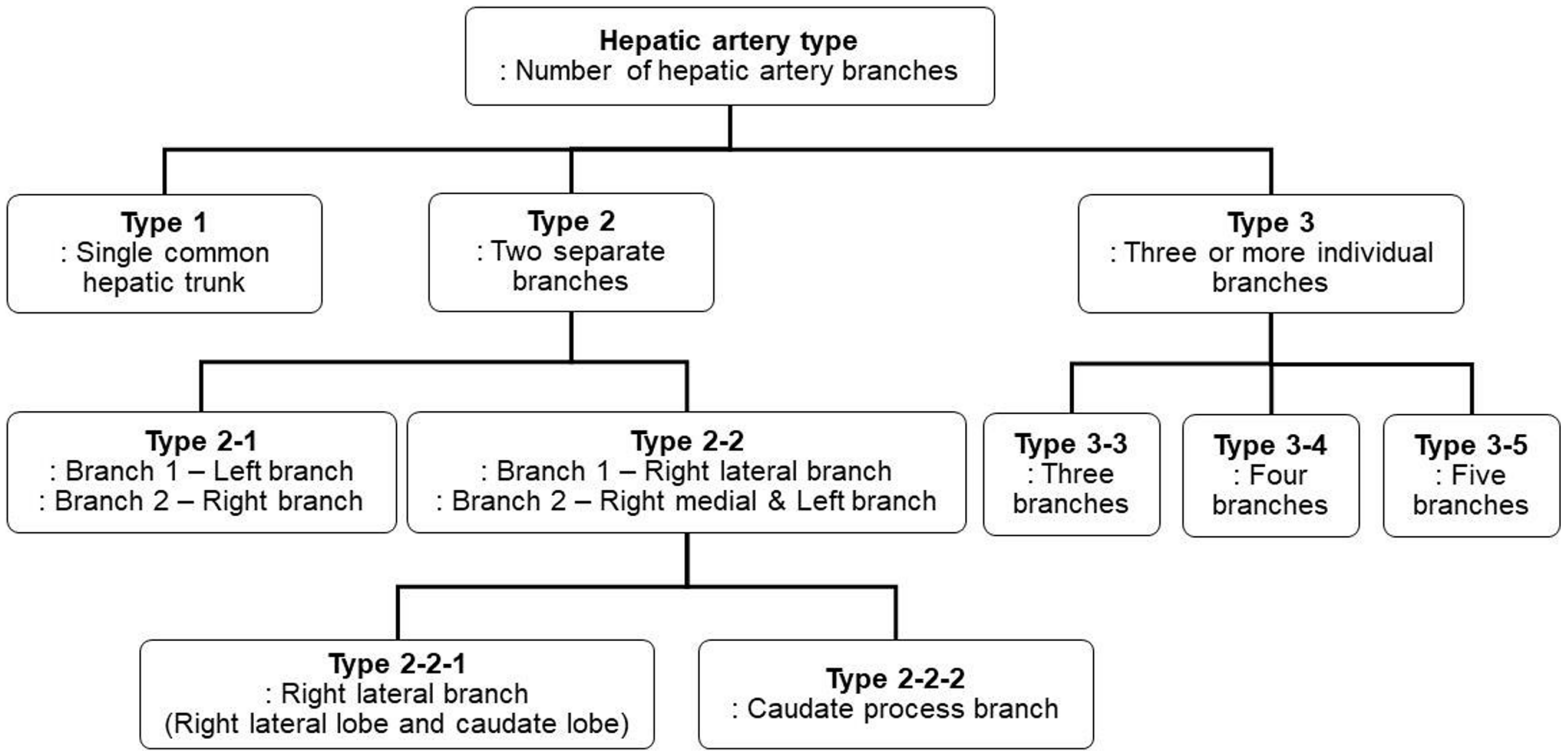
The schematic diagram of classification of canine hepatic artery branching types. According to the previous studies, hepatic artery ramification types were categorized into three major types. Type 2 was further subdivided into two subtypes, Type 2-1 and Type 2-2, based on which lobe each branch runs to, and Type 2-2 was then further divided into Type 2-2-1 and Type 2-2-2 based on the presence of caudate process branch. Type 3 was subdivided into Type 3-3, Type 3-4, and Type 3-5 based on the number of branches.

**Figure 2:**
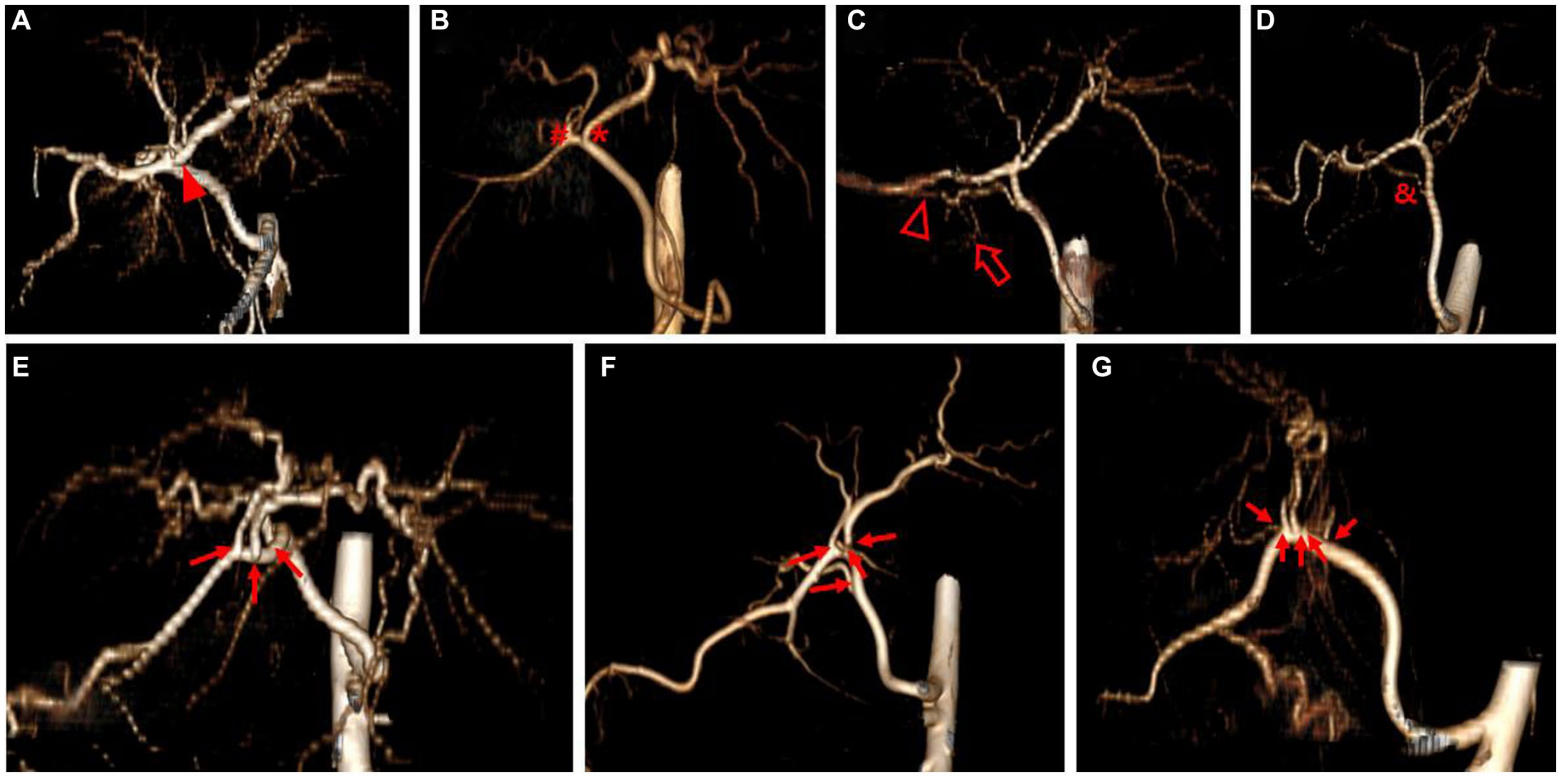
3D volume rendering image representing each hepatic artery branch type. Type 1 **(A)** shows single common trunk (arrowhead). Type 2 **(B–D)** is divided into two separate branches; Type 2-1 **(B)** consists of the left branch (asterisk) and the right branch (hashtag), Type 2-2-1 **(C)** has the right lateral branch that runs to both the right lateral lobe (hollow arrowhead) and the caudate process (hollow arrow), and Type 2-2-2 **(D)** has caudate process branch (ampersand). Type 3 **(E–G)** shows more than three branches (arrow) arising from hepatic artery; Type 3-3 **(E)** shows three branches, Type 3-4 **(F)** shows four branches, and Type 3-5 **(G)** shows five branches. The best side to display the hepatic artery branches was chosen for each image.

Three-dimensional volume rendering images were used to analyze the branching pattern of hepatic artery. According to the previous studies (17–20), the hepatic artery ramification types were divided into three major types based on the number of main branches branching from hepatic artery. In the case of branching into a single common trunk from the hepatic artery, it was classified as type 1, branching into two separate branches was classified as type 2, and branching into three or more branches was classified as type 3. Type 2 was subdivided into type 2-1 and type 2-2 according to whether the two branches were divided into left and right branches or right lateral branch and right medial and left branch, respectively, and type 2-2 was classified as type 2-2-1 when the right lateral branch run into the right lateral lobe and the caudate process, and type 2-2-2 when the first branch was caudate process branch. Type 3 was classified into type 3-3 when the number of branches was 3, 3-4 for 4, and 3-5 when the number of branches was 5 or more. Figure 1 depicted a schematic representation of the hepatic artery branch type of this study, and 3D representations of each type of hepatic artery branch were shown in Figure 2.

The celiac artery diameter was measured immediately after branching from the aorta and the hepatic artery diameter was measured just before the first main branch, with a window width of 600 and window level of 300 in transverse image.

### 2.4 Hepatic mass group

Dogs that underwent CT scans for liver-related problems and masses observed in the hepatic parenchyma were included in hepatic tumor group, and dogs with hepatic vessel anomalies such as portosystemic shunt or chronic hepatic diseases such as cirrhosis involving the entire liver were excluded. In the hepatic tumor group, the maximum diameter and number of masses, the number and division of affected lobes, and whether the blood vessels to the mass are visible were assessed with a window width of 400 and window level of 60 in transverse image. In addition, in patients who underwent the diagnostic tests for the hepatic masses, the method and final diagnosis were collected.

### 2.5 3D model development

The 3D models were custom-made by the 3D technology company (Medical IP company, Seoul, Korea), made of high-transparent silicone. One dog from each group was selected as the subject for the 3D models. The dog with the celiac and hepatic artery diameters closest to the mean value and with the most common vascular pattern type was chosen for the creation of the 3D model of the normal canine hepatic artery. The vessels less than 1 mm were regarded as 1 mm, due to the minimum feasible diameter of 3D printing. For the 3D hepatic mass model, a dog was selected in which multiple lesions involved multiple hepatic lobes, were adjacent to the diaphragm or large blood vessels where surgical removal is challenging, and the inflow of arteries into the tumor was clearly identified in the CT arterial phase.

### 2.6 Transarterial selection and embolization simulation in 3D models

The simulation was performed under fluoroscopic guidance (Veradius Unity; Philips, Netherlands). The part of the 3D models that corresponds to the femoral artery was punctured with a 20G needle or IV catheter and cannulated with 5Fr introducer sheath. 0.035 in angled guidewire and 5Fr bumper-tip catheter were inserted into the aorta and anchored at the celiac artery. The guidewire was removed and the 0.018 in microguidewire and 2.4Fr microcatheter were inserted into the common hepatic artery through 5Fr catheter, then placed into the targeted lobar artery. Gelatin sponge particle (GSP, Spongostan Standard; Ethicon, United States) was used as embolizing material mixed with contrast media, iohexol (Omnipaque, GE healthcare, Austria). After embolization, contrast media was injected for identification of occlusion of targeted arteries. Due to the wide vessel diameter in the hepatic mass model, only a 5Fr bumper-tip catheter was employed, without microcatheter.

### 2.7 Statistical analysis

The patient information and the measured data were recorded using a Microsoft Excel spreadsheet (Microsoft Excel 2016, Microsoft Corporation, Redmond, WA, United States), and the mean, SD, and range of the data were analyzed and used as descriptive statistics. Two commercially available statistical software, SPSS 21.0 (IBM SPSS Statistics, Chicago, IL, United States) and Prism 9.0 (GraphPad Software Inc., San Diego, CA, United States), were used for all statistical analyses and statistical figures. The measured data were checked for normal distribution using the Kolmogorov–Smirnov test with the SPSS software. Correlation analyses were performed for the weight and the celiac and hepatic arteries using Prism 9.0, Pearson correlation analyses were applied if the results were normally distributed, and Spearman correlation analyses were used if the results were not distributed normally. *p* < 0.05 was set for statistical significance.

## 3 Results

### 3.1 Normal hepatic group

**Figure 3:**
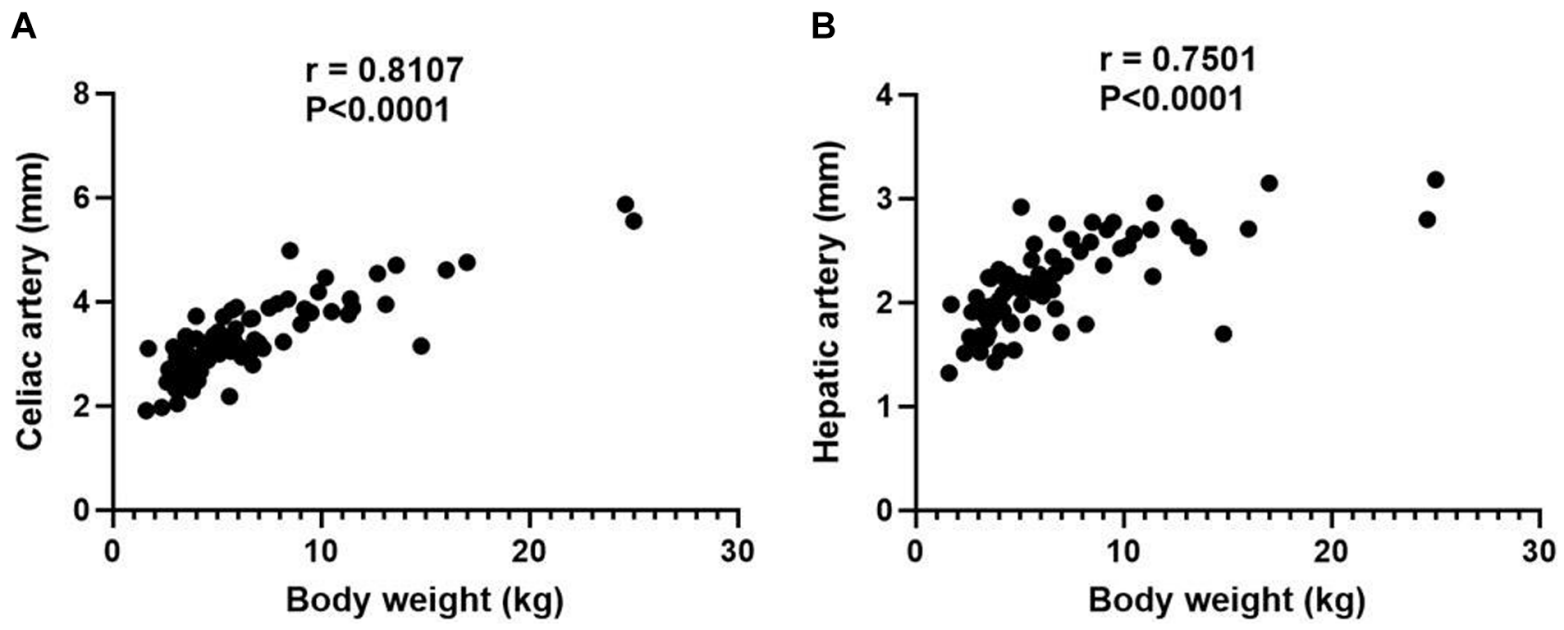
Scatter plot illustrating the relationship between body weight and the diameters of celiac artery **(A)** and hepatic artery **(B)**. *p* value of 0.05 considered significant, and both results demonstrate statistical significance with *p* < 0.0001.

In normal hepatic group, 84 dogs were included and the mean body weight and standard deviation (SD) was 6.47 ± 4.44 kg, ranging from 1.6 to 25.0 kg. The characteristics of dogs are summarized in Table 1. The mean diameter and SD of celiac and hepatic artery was 3.28 ± 0.77 mm (range; 1.91–5.87 mm) and 2.14 ± 0.43 mm (range; 1.32–3.18 mm) for each. Both results did not follow the normal distribution; spearman correlation analyses were adopted and demonstrated the significant correlation between the body weight and the diameter of celiac artery (*rs* = 0.8107, *p* < 0.0001), and the body weight and the diameter of hepatic artery (*rs* = 0.7501, *p* < 0.0001; Figure 3).

**Table 1:** Characteristics of 84 normal hepatic artery group dogs.

	Normal hepatic artery group dogs (*n* = 84)
Age (years)	10.14 ± 3.14; 1–17
Body weight (kg)	6.47 ± 4.44; 1.6–25.0
Sex (*n*)	Castrated male (38), Spayed female (29), Intact male (4), Intact female (13)
Breed (*n*)	Maltese (26), Poodle (12), Yorkshire terrier (6), Spitz (4), Schnauzers (4), Shih tzu (4), Mixed (3), French bulldog (3), Dachshund (3), Cocker spaniel (3), Pomeranian (3), Welsh corgis (2), Pekingese (2), Chihuahua (2), Jindo and Jindo mixed (2), Soft coated wheaten terrier (1), Scottish terrier (1), Chow chow (1), Border collie (1), and Miniature pinscher (1)
Reason for CT	Splenic mass (23), mammary gland tumor (10), GI mass (9), adrenal mass (7), perineal mass (4), female reproductive system mass (4), skin mass (3), renal cyst (2), UB mass (2), pulmonary mass (2), abdominal mass (2), herniation (2), NRF (2), bile duct dilation (1), bite wound (1), tarsal mass (1), thyroid mass (1), GB edema (1), IVDD (1), enlarged lymph node (1), MCT (1), pancreatitis (1), pleural mass (1), thrombus (1), and cholangiohepatitis(1)

Age and body weight represent mean ± standard deviation; range.

GB, Gallbladder; GI, Gastrointestinal; IVDD, Intervertebral disk disease; MCT, Mast cell tumor; NRF, No remarkable findings; and UB, Urinary bladder.

The most common hepatic artery type was type 2-2-1, which included 36 dogs, followed by type 2-1 with 15 dogs. There were 14 dogs with type 3-3, 7 with type 1 and type 2-2-2 for each, 3 with type 3-4, and 2 with type 3-5 (Table 2).

**Table 2:** Prevalence of each hepatic artery branching type in 84 normal hepatic artery group dogs.

Hepatic artery branching type	Type 1	Type 2			Type 3		
		Type 2-1	Type 2-2		Type 3-3	Type 3-4	Type 3-5
			Type 2-2-1	Type 2-2-2			
Number of cases	7	15	36	7	14	3	2

### 3.2 Hepatic mass group

Sixty-five dogs were included in the hepatic mass group, and the mean maximum mass diameter and SD was 73.84 ± 38.03 mm, ranging from 15.8 to 196.2 mm. Artery flow into the mass was identified in 58 dogs. The detailed characteristics are summarized in Table 3.

**Table 3:** Characteristics of 65 hepatic mass group dogs.

	Hepatic mass group dogs (*n* = 65)
Age (years)	11.7 ± 2.51; 5–18
Body weight (kg)	6.71 ± 6.36; 1.94–35.3
Sex (*n*)	Castrated Male (33), Spayed Female (23), Intact Female (5), and Intact Male (4)
Breed (*n*)	Maltese (17), Shih tzu (14), Mixed (10), Poodle (6), Yorkshire terrier (5), Dachshund (2), Scottish terrier (1), Samoyed (1), Spitz (1), Schnauzers (1), Jindo(1), Pomeranian(1), Miniature pinscher (1), Labrador retriever (1), Bichon frise (1), Beagle (1), and Alaskan malamute (1)
Maximum mass diameter (mm)	73.84 ± 38.03; 15.8–196.2
Artery flow into mass	Y (58)/N (7)

Age, body weight, and maximum mass diameter represent mean ± standard deviation; range.

N, No; Y, Yes (In case of the inflow of blood vessels into the tumor was confirmed in arterial phase, the arterial flow into mass was recorded as Y, and otherwise as N).

Twenty-five of this group had solitary lesions, and 11 had multifocal lesions in the single lobe. Among them, 17 had a maximum lesion size of 7 cm or more, and 19 had a size less than 7 cm. Of the 29 dogs with multifocal lesions in multiple lobes, 14 had lesions on two lobes, eight on three lobes, and seven on four or more lobes. Nineteen of them had a maximum lesion size of 7 cm or more, and 10 had a size less than 7 cm. When divided into groups depending on the three divisions of hepatic lobes, 23 had lesions only in the left division, the left medial, and left lateral lobe, and therefore, the remaining 42 were found to have at least one lesion on the right and central division, the quadrate and right medial lobe, the right lateral and caudate lobe.

Of the total, the histological diagnoses were identified in 28 dogs, which were hepatocellular carcinoma (*n* = 13), hepatocellular adenoma (*n* = 8), nodular hyperplasia (*n* = 4), and hemangiosarcoma, biliary cyst adenoma, and lymphoma (*n* = 1), and most of them were diagnosed through surgical biopsy (*n* = 20), followed by biopsy and FNA (*n* = 4).

### 3.3 Development of 3D canine artery model with normal hepatic artery and hepatic mass

**Figure 4:**
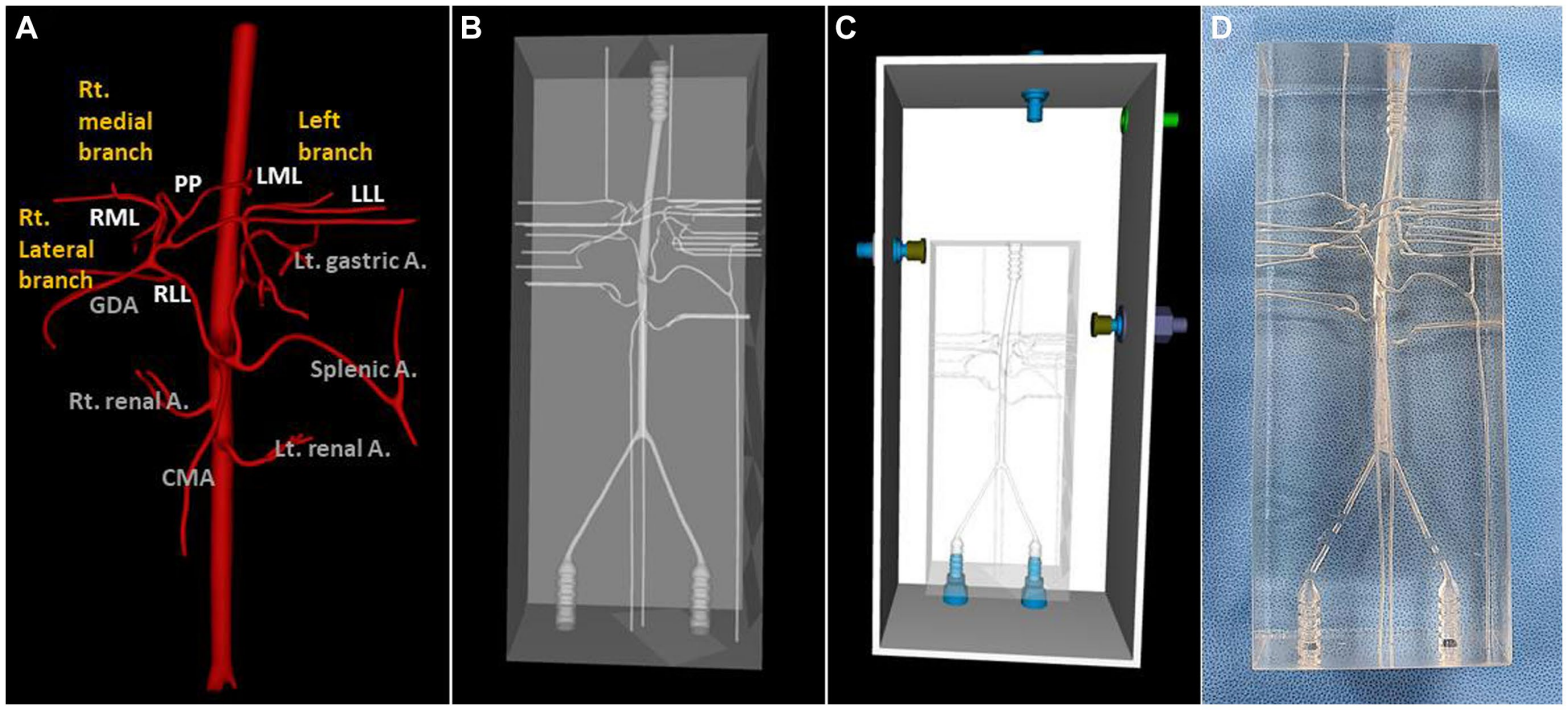
3D-modeling virtual prototype (VP) and the real model image of normal canine arteries of type 2-2-1. Ventro-dorsal view of hepatic artery VP **(A)**, model VP **(B)**, total model VP including outer acrylic pool **(C)**, and the real model **(D)**.

**Figure 5:**
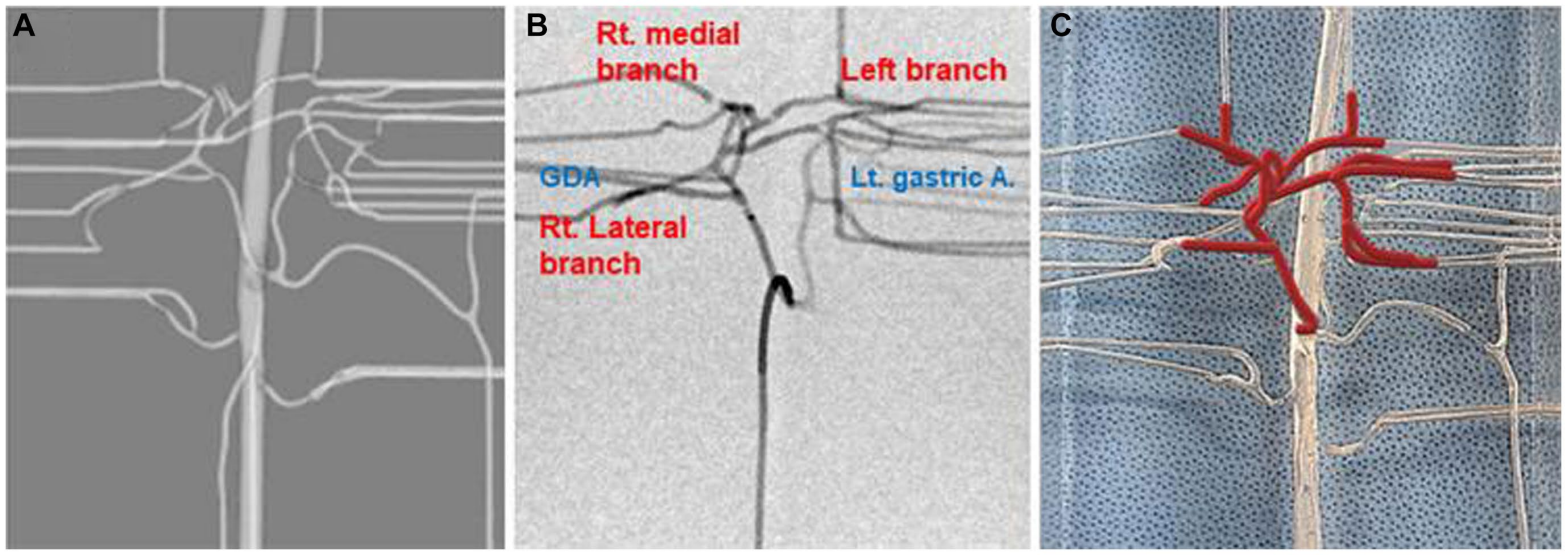
Virtual prototype image for the hepatic artery **(A)**, contrast-enhanced fluoroscopic image **(B)**, and photograph of model illustrating of hepatic arteries in red lines **(C)**.

A dog that weighed 4.38 kg, had a celiac and hepatic artery diameter of 3.21 and 2.21 mm for each and had artery type 2-2-1 who had CT for splenic mass was selected for the creation of a 3D normal hepatic artery model. All hepatic arteries were reflected in the actual model, except the caudate process artery, because the connection between the caudate process artery and the right lateral lobe artery was not applied during 3D modeling. Figures 4, 5 illustrate the 3D modeling process and the realistic image of the model, and Figure 5 emphasizes the region of the hepatic artery.

**Figure 6:**
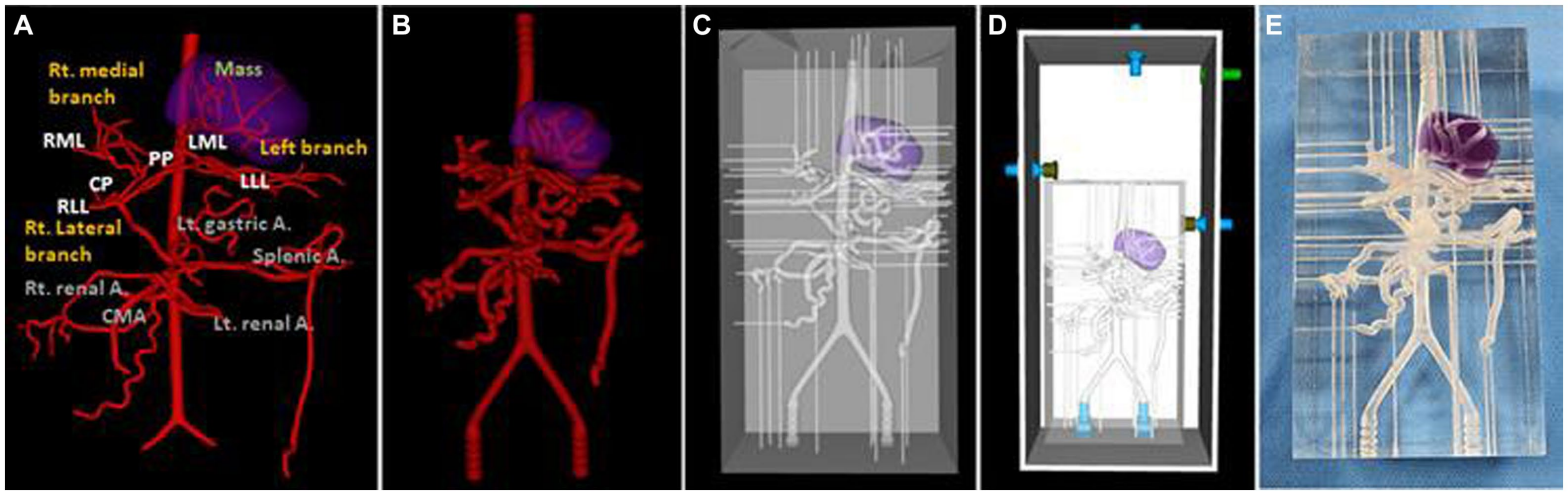
Ventro-dorsal view of 3D-modeling virtual prototype (VP) of canine hepatic mass and arteries **(A)**, artery VP for actual model with duplicated vessel size **(B)**, model VP **(C)**, total model VP including outer acrylic pool **(D)**, and the photograph of model **(E)**. The mass lesion is indicated by purple area.

For the 3D hepatic mass model, a dog with multiple lesions existing in three hepatic lobes, of which the largest tumor was located in the left medial lobe adjacent to the diaphragm with the inflow of arteries clearly observed, was selected, and only the largest tumor was visualized in the model. The vascular structure of the hepatic tumor was more complicated due to the development of arteries, and insertion of the catheter was judged to be difficult due to the frictional force with the high-transparent silicone used in the 3D model, so the diameter of the whole blood vessels was expanded to double. Figure 6 showed a realistic rendering of the model along with the 3D modeling process.

**Figure 7:**
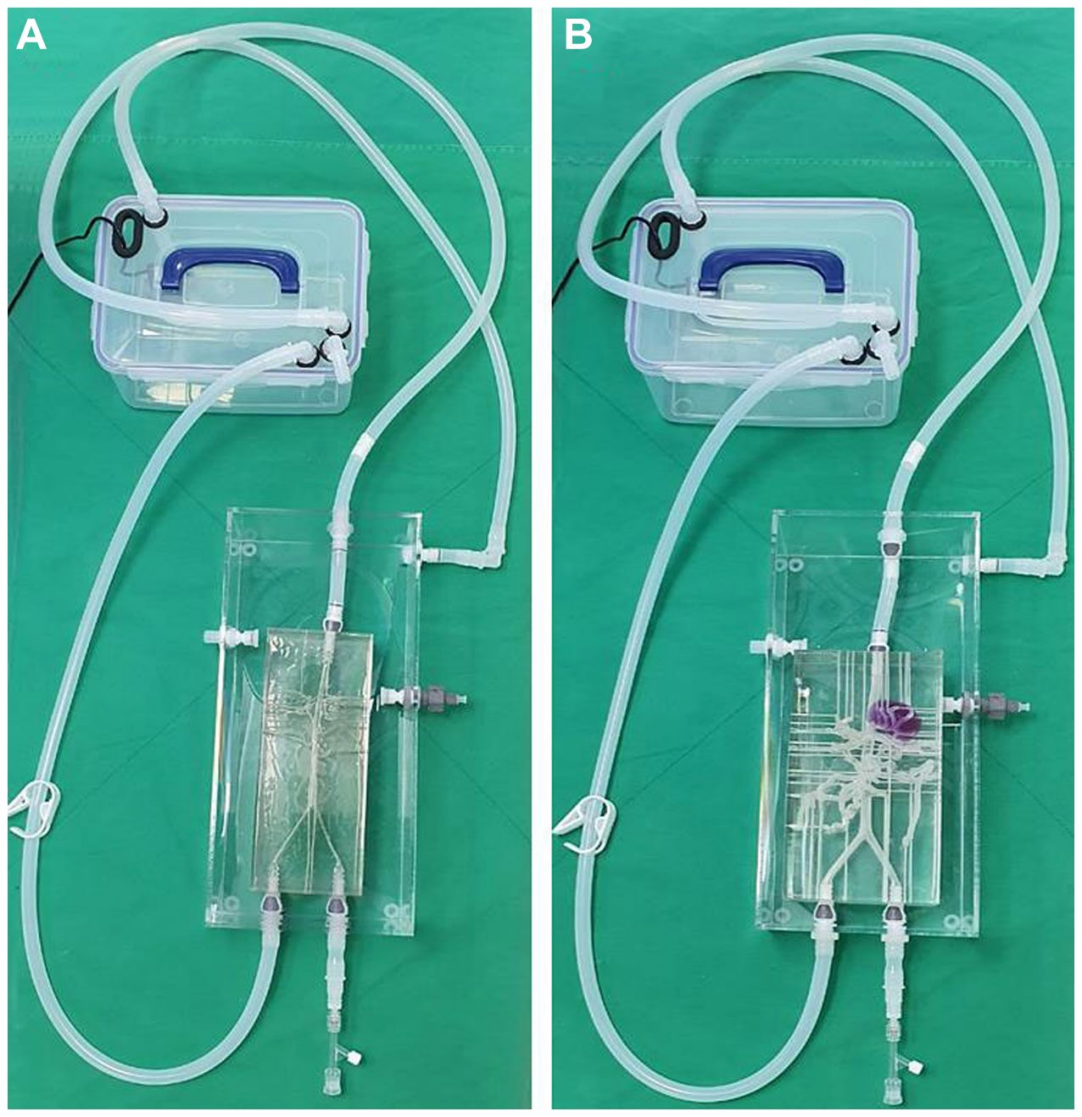
Photograph of the 3D printed normal canine artery model **(A)** and hepatic mass model **(B)** connected to the acrylic pool and combined with the flow pump using silicone hoses. Both models share the same acrylic pool, flow pump, and silicone hoses.

The two models shared the same acrylic pool, and the silicone hoses were connected to an external pump so that water flows from the descending aorta to the iliac artery direction in the same way as the actual blood flow (Figure 7). External outflow channels with a diameter of about 1 mm were installed for each blood vessel to discharge water flow into the artery to the outside pool. The water that flows into the pool was then designed to enter the pump again through a silicone hose.

**Figure 8:**
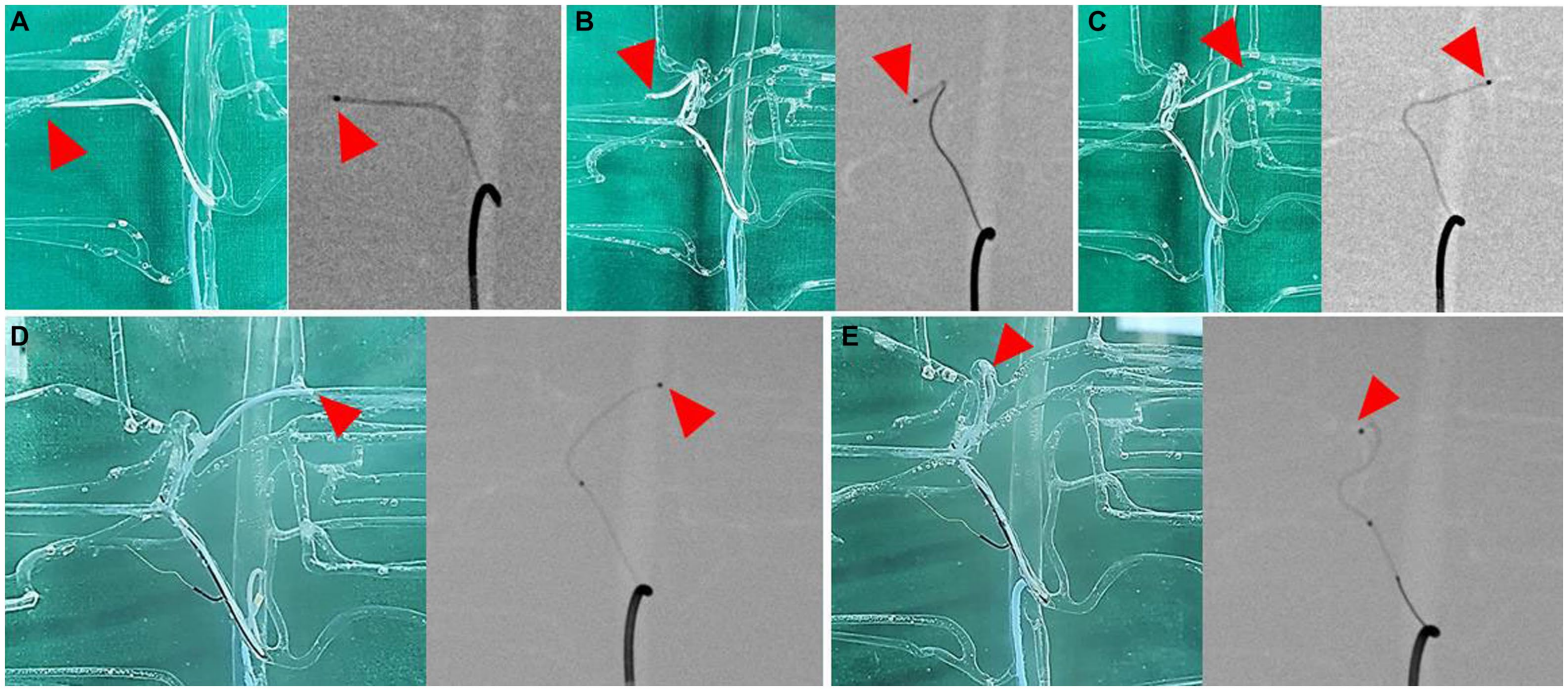
Selection of hepatic artery branches, 3D printed model (left) and fluoroscopic image (right) for each image. Superselection of right lateral lobe artery **(A)**, right medial lobe artery **(B)**, left lateral lobe artery **(C)**, left medial lobe artery **(D)**, and papillary process artery **(E)**. Arrowhead indicates the tip of microcatheter.

**Figure 9:**
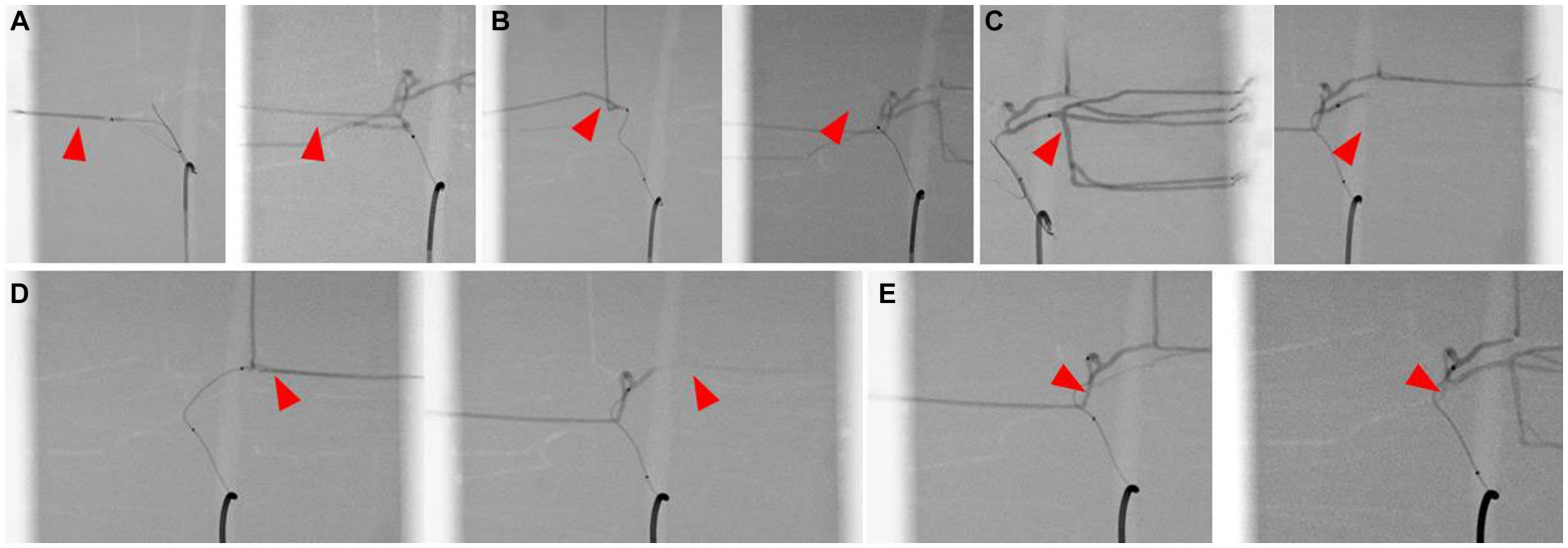
Simulation of hepatic artery embolization of each lobes, contrast-enhanced fluoroscopic image of pre-embolization (left) and post-embolization (right) of right lateral lobe artery **(A)**, right medial lobe artery **(B)**, left lateral lobe artery **(C)**, left medial lobe artery **(D)**, and papillary process artery **(E)**. Arrowhead indicates the selected lobar artery, and absence of the contrast agent in the post-embolization images suggests the embolization of the artery.

**Figure 10:**
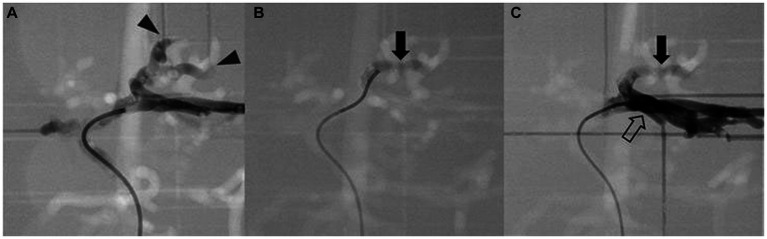
Simulation of arterial embolization of hepatic mass. Contrast-enhanced fluoroscopic image of pre-embolization **(A)** shows the contrast media flow into the arteries of mass (arrowhead), embolizing material mixed with contrast media (arrow) is located at the insertion of the mass **(B)**, and post-embolization image **(C)** shows the contrast media is unable to enter the embolized arteries of the tumor then returns to adjacent left lateral arteries (hollow arrow).

The catheter insertion and embolization were performed into all possible hepatic arteries in the normal model. Under fluoroscopic guidance and contrast media injection, it was verified that the desired artery had been selected, the blood flow was normal prior to embolization, and the blood arteries were embolized successfully (Figures 8, 9). The same simulation was performed in the hepatic mass model, and embolization was performed at the entrance of the artery entering the tumor (Figure 10).

## 4 Discussion

In this study, the normal hepatic artery structure of dogs of various weights and breeds was analyzed, and the most common type of hepatic artery was type 2-2-1, the same as in the previous studies (17–20). The diameters of the celiac and hepatic arteries were identified as 3.28 ± 0.77 and 2.14 ± 0.43 mm for each in this study with body weight ranging from 1.6 to 25.0 kg, and it was found that the celiac and hepatic arteries had a significant correlation with weight. In the hepatic tumor group, 44.6% (29/65) of the cases had tumors in multiple lobes and 64.6% (42/65) had at least one lesion in central or right division. We made two 3D hepatic artery models for TAE simulation. In the normal hepatic artery group, a dog with the diameter of celiac artery and hepatic artery closest to the mean values of 3.28 and 2.14 mm, respectively, and with a most common branch type of the hepatic artery of 2-2-1 was selected for normal hepatic artery model. In the hepatic tumor group, a dog with multifocal lesions adjacent to the diaphragm, which are difficult to operate, with arterial inflow into the tumors clearly observed in CT arterial phase was selected for 3D model production. Using these models, placement of microcatheters and embolization of each lobar artery was possible in both the normal hepatic artery model and the hepatic mass model, even when angiogenesis and mass effect further complicated the arteries.

In this study, only 38.5% (25/65) of the hepatic mass group had a solitary liver lesion, and 44.6% (29/65) of the group were found to have multifocal lesions in multiple lobes, for which surgical resection is not recommended and TAE can be the effective treatment option. Even with the solitary lesion, that prognosis is known to be good with surgery, the difficulty and outcome of lobectomy differs from the division because of the anatomy of the liver (30). Lobectomy of the left division is usually simpler, and the right or central division lobectomy shows more complications and mortalities (8, 30), TAE can be applied to solitary massive liver mass in the right and central division more effectively than surgery. Therefore, we tried to choose a patient with multiple lesions and the largest mass located in the right or central division, however, for accurate implementation of blood vessels, CT images with clearly obtained arteries were needed, so we chose the patient with a lesion in left medial lobe, but close to diaphragm instead, for the hepatic mass model. Likewise, we chose a patient that is thought to be difficult to get surgery, because the exact criteria for where TAE is more effective than surgical resection are unknown in veterinary medicine. In human medicine, there are many criteria for the selection of adequate treatment for liver mass, such as BCLC stage, Child-Pugh Score, and ECOG score (PS score) (31–33). But the causes and types of liver tumors are different between humans and dogs, like hepatic tumors are commonly driven by cirrhosis associated with the hepatitis B and C virus, and heavy alcohol consumption and nodular and diffuse types are more common in human (34, 35), it is difficult to apply the same criteria in veterinary medicine. For example, hepatic masses more than 7 cm are classified as an indication of poor prognosis of TACE in humans (31–33), but according to previous case reports in dogs, TAE was successfully applied in dogs with maximum mass size more than 7 cm (36, 37). New TAE and TACE criteria that are suitable for dogs will therefore be needed.

There are some limitations of the models in this study. First, due to the nature of high-transparent silicone used for the models, the friction force was higher than that of the actual blood vessels, and even though catheters suitable for the diameter of the blood vessels were chosen and used during the simulation, more force was needed as the number of curvatures of vessels increased, which had an impact on the increase of friction force. The decision to use the material for the model with this restriction was made due to cost-effectiveness as well as current technological constraints, such as those relating to manufacturing processes and the inability to coat the interior of small vessels. Instead, it was possible to use semi-permanently by improving durability to reflect the elastic properties of blood vessels by adopting the current silicone material. To create a model using a novel material that reflects the properties of real vascular endothelium and also has good durability, more research about newly developed materials and attempts to upgrade the models will be required.

Secondly, in this study, the caudate process artery was not reflected in the normal hepatic artery model. The discontinuity of caudate process artery from the right lateral lobe artery was seen in the DICOM image of the arterial phase due to the tiny blood vessel width and characteristics of the terminal position, which can be taken into account to some degree when evaluating the image, but it was not accurately reflected in the creation of the 3D model. However, when blood vessels are omitted, it can be improved by providing clear instructions and careful observation of terminal blood vessels that are prone to omission when re-manufacturing the model.

Finally, an external pump and hoses with clips were used to determine the direction and speed of the blood flow. However, poorer circulation was seen the farther away the blood vessels were from the aorta, and in the tumor model, this was because of the intricate blood vessel structure and the fact that the external outflow tracts were made at roughly 1 mm for embolization, which is relatively tiny and can disturb the discharge of water out of vessels. By changing the way blood exits the artery and replacing the pump, it can be made to resemble actual blood flow as closely as possible.

In conclusion, we analyzed the hepatic artery structures in dogs including small and toy breed dogs ranging from 1.6 to 25.0 kg those were not included in previous studies, and the result showed the most common artery branch type was the same as previous studies. Also, the celiac and hepatic artery diameters, which are important for selecting the size of the catheter in TAE procedure, have a positive correlation to the weight of the dog. In the hepatic mass group, about 45% of them had multifocal lesions in multiple liver lobes, which TAE procedure can be effective. We create 3D canine artery models with and without tumors through the course of this study that can simulate TAE procedures repeatably whether it will still be necessary to produce upgraded models by optimizing the material of models, accurate reflection of the vascular structure, and actual blood flow. It is anticipated that the development of these models will allow for the further activation of interventional procedures in veterinary medicine. We expect that practitioners can increase their proficiency by practicing selection on hepatic blood vessels through the models created in this study and considering the traits of animals that need general anesthesia even for interventional procedures, the increased proficiency can also shorten the duration of anesthesia, which is crucial for the postoperative prognosis. Moreover, it may be the answer to the ethical dilemma by replacing the use of experimental animals for practicing procedures.

## Data availability statement

The original contributions presented in the study are included in the article/supplementary material, further inquiries can be directed to the corresponding author.

## Ethics statement

Ethical approval was not required for the study involving humans in accordance with the local legislation and institutional requirements. Written informed consent to participate in this study was not required from the participants or the participants’ legal guardians/next of kin in accordance with the national legislation and the institutional requirements. Ethical approval was not required for the study involving animals in accordance with the local legislation and institutional requirements because this study was conducted as a retrospective study.

## Author contributions

MO: Writing – original draft. JB: Writing – original draft. YL: Writing – original draft. ML: Writing – original draft. SK: Writing – original draft. UK: Writing – original draft. JP: Writing – original draft. JH: Writing – original draft. JC: Writing – original draft. BK: Writing – original draft. HY: Writing – original draft. NL: Writing-original draft. DC: Writing – original draft.
